# Enterotoxigenic *Bacteroides fragilis* Induces Host Genotype-Specific Colonic Epithelial and Immune Responses in Mice

**DOI:** 10.1093/infdis/jiag247

**Published:** 2026-05-06

**Authors:** Taylor Southward, Yongbao Zhuang, Ada Tam, Hana Minsky, Jacqueline Ferri, John Michel, Shaoguang Wu, Xinqun Wu, Tatianna Larman, Franck Housseau, James R White, Cynthia L Sears, Jessica Queen

**Affiliations:** Division of Infectious Diseases, Department of Medicine, Johns Hopkins University School of Medicine, Baltimore, Maryland, USA; Division of Infectious Diseases, Department of Medicine, Johns Hopkins University School of Medicine, Baltimore, Maryland, USA; Department of Oncology, Johns Hopkins University School of Medicine, Baltimore, Maryland, USA; Division of Infectious Diseases, Department of Medicine, Johns Hopkins University School of Medicine, Baltimore, Maryland, USA; Department of Oncology, Johns Hopkins University School of Medicine, Baltimore, Maryland, USA; Department of Oncology, Johns Hopkins University School of Medicine, Baltimore, Maryland, USA; Division of Infectious Diseases, Department of Medicine, Johns Hopkins University School of Medicine, Baltimore, Maryland, USA; Division of Infectious Diseases, Department of Medicine, Johns Hopkins University School of Medicine, Baltimore, Maryland, USA; Department of Pathology, Johns Hopkins University School of Medicine, Baltimore, Maryland, USA; Department of Oncology, Johns Hopkins University School of Medicine, Baltimore, Maryland, USA; Bloomberg~Kimmel Institute, Johns Hopkins University School of Medicine, Baltimore, Maryland, USA; Resphera Biosciences, Baltimore, Maryland, USA; Division of Infectious Diseases, Department of Medicine, Johns Hopkins University School of Medicine, Baltimore, Maryland, USA; Department of Oncology, Johns Hopkins University School of Medicine, Baltimore, Maryland, USA; Bloomberg~Kimmel Institute, Johns Hopkins University School of Medicine, Baltimore, Maryland, USA; Division of Infectious Diseases, Department of Medicine, Johns Hopkins University School of Medicine, Baltimore, Maryland, USA; Department of Oncology, Johns Hopkins University School of Medicine, Baltimore, Maryland, USA

**Keywords:** bacteroides, microbiome, colorectal cancer, colitis, oncogene

## Abstract

**Background:**

Colorectal cancer pathogenesis involves complex interactions between multiple risk factors including somatic mutations and microbial dysbiosis. A number of individual microbiota members have been implicated in colorectal cancer, including enterotoxigenic strains of *Bacteroides fragilis* (ETBF). Enterotoxigenic *Bacteroides fragilis* promotes inflammation in mouse models, which has been mechanistically linked to colon tumorigenesis. We hypothesized that ETBF would promote distinct patterns of colonic damage and inflammation in mice expressing different oncogenic mutations.

**Methods:**

Mice expressing mutations in the *Apc* tumor suppressor gene or the *BRAF* or *Kras* oncogenes were colonized with ETBF to induce acute colitis. Seven days after colonization, tissues and stools were collected to assess for colonization, epithelial damage, and local and systemic immune responses.

**Results:**

Despite uniform colonization of ETBF across all genotypes and some common features of colitis across groups, *Apc*, *BRAF*, and *Kras* mutations were associated with distinct patterns of colonic epithelial cell injury and goblet cell loss in response to ETBF. RNA sequencing analysis revealed varied transcriptional profiles based on mouse genotype and colon region. Flow cytometry of intra-epithelial leukocytes revealed differential recruitment of myeloid cells based on oncogenic mutation. In particular, mutant BRAF expression was uniquely associated with more systemic inflammation, resistance to goblet cell loss, an interferon gamma gene signature, and recruitment of a macrophage-like polymorphonuclear myeloid-derived suppressor cell (PMN-MDSC) population in the midproximal colon.

**Conclusions:**

Enterotoxigenic *Bacteroides fragilis* promotes acute colitis in mice expressing different oncogenic mutations, but with distinct patterns of colonic epithelial cell damage and inflammation dependent on host oncogene context.

## Teaser

We investigated how expression of different oncogenic mutations altered host responses to enterotoxigenic *Bacteroides fragilis* (ETBF) in a mouse model of acute colitis. We identified distinct epithelial and immune responses to ETBF dependent on host oncogene context.

Data support a key role for microbiome dysbiosis in colorectal cancer (CRC), a leading cause of cancer morbidity and mortality. Colorectal cancer is a heterogeneous disease, classified into consensus molecular subtypes with distinct oncogene expression, DNA mutation, epigenetic methylation, immune cell infiltration, prognosis, and response to immunotherapy [1]. Loss of function mutations in the tumor suppressor gene *APC* and activating mutations in the oncogenes *KRAS* and *BRAF* are common in CRC, seen in approximately 80%, 40%, and 10% of sporadic colon tumors, respectively [2]. However, little is known about the intersection of the microbiota with the molecular subtypes or their associated oncogenes, although this has profound implications for understanding disease mechanisms and therapeutic response.

*Bacteroides fragilis* is a ubiquitous human symbiotic organism, a subset of which harbor the *B. fragilis* toxin (BFT), a zinc metalloprotease that triggers E-cadherin cleavage in the colon epithelium, leading to β-catenin activation that promotes proliferation and migration [3, 4]. Colon enterotoxigenic *B. fragilis* (ETBF) strains are enriched in patients with CRC [2, 5–7]. While ETBF-colonized multiple intestinal neoplasia (Min) mice expressing *Apc^MinΔ716/−^* have been well-studied [8–10], adding *BRAF^V600E^*, but not *Kras^G12D^*, to the Min ETBF model promoted a distinct tumor phenotype and tumor immune microenvironment (TiME), characterized by a new midproximal locus of serrated-like tumors, disrupted mucus bilayer, an IFNγ signature, CD8+ T cell and granulocytic myeloid-derived suppressor cell (PMN-MDSC) infiltration, and responsiveness to PD-L1 blockade [11]. The contrasting tumor phenotypes in these mice are striking, as *RAF* is immediately downstream of *RAS* in the mitogen-activated protein kinase (MAPK) signaling pathway. In human CRC, *BRAF* and *KRAS* mutations are associated with distinct molecular and clinical features [11]. Mutant *BRAF* is associated with CpG island methylation, microsatellite instability, proximal disease, lymphocyte infiltration, and a mucinous phenotype, and is most common in CMS1 tumors. *KRAS* mutations, meanwhile, are often associated with distal tumors with microsatellite stability and are overrepresented in CMS3 tumors. Importantly, both *BRAF* and *KRAS* mutations are frequently observed in premalignant lesions, highlighting their role in tumor initiation [12, 13].

Herein, we investigate the early host response to ETBF, finding that this cancer-associated microbe promotes distinct pro-carcinogenic mechanisms that involve diverse patterns and phenotypes of myeloid cell recruitment and epithelial and immune gene expression, dependent on host oncogene context.

## METHODS

### *Bacteroides fragilis* Colonization Model

All animal protocols were approved by the Johns Hopkins University Animal Care and Use Committee. Male and female mice were housed in a specific-pathogen-free facility with a 12-h light/dark cycle and fed standard chow and water ad libitum. Mouse strains are listed in Supplementary Table 1. Strain sources included wildtype C57BL/6J (WT; JAX:000664), *Apc^MinΔ716−^* (Min; Dr. David Huso, Johns Hopkins University), Loxp-flanked *BRAF^V600E^* (B6.129P2(Cg)-Braf^tm1Mmcm^/J; JAX:017837), Loxp-flanked *Kras^G12D^* (B6.129S4-Kras^tm4Tyj^/J; JAX:008179), and leucine rich repeat containing G protein-coupled receptor 5 (Lgr5) CreERT2 knock-in mice (B6.129P2-Lgr5^tm1(cre/ERT2)Cle^/J; JAX:008875). Lgr5 CreERT2 knock-in mice were crossed with Loxp-flanked *BRAF^V600E^* and *Kras^G12D^* mice, respectively, to yield *BRAF^V600E^Lgr5^tm1(Cre/ERT2)Cle^* (BL) or *Kras^G12D^Lgr5^tm1(Cre/ERT2)Cle^* (KL) mice. Recombination in mice bearing Lgr5^Cre^ was induced with tamoxifen, as previously described, at 4–6 weeks of age [14].

To limit cage-specific changes to the gut microbiota, mice of different genotypes were co-housed for all experiments. Mice were treated with streptomycin (5 mg/mL) and clindamycin (0.1 mg/mL) ad libitum in drinking water for 5 days, and then inoculated by orogastric gavage with ETBF strain 86–5443–2-2 or phosphate-buffered saline (PBS) for sham controls. To confirm colonization, fecal DNA was isolated using the Zymo Quick-DNA Fecal/Soil Microbe Kit (Zymo Research, D6011), and analyzed by quantitative PCR for the *B. fragilis* 16S rRNA gene (F: TCRGGAAGAAAGCTTGCT, R: CATCCTTTACCGGAATCCT, probe: 5′HEX-ACACGTATCCAACCTGCCCTTTACTCG-3′BHQ1) and *bft* (F: GGATAAGCGTACTAAAATACAGCTGGAT, R: CTGCGAACTCATCTCCCAGTATAAA, probe: 5′FAM - CAGACGGACATTCTC - 3′ NFQ-MGB) [15, 16]. Colonization was confirmed for a subset of mice by enumeration of colony forming units (CFUs).

### Tissue Collection, Fixation, and Histology

For histopathology and immunohistochemistry, colons were flushed with PBS, formalin-fixed (10%), and paraffin-embedded. Histological assessments were performed by a blinded pathologist using a comprehensive scoring system consisting of several parameters of inflammation: inflammatory infiltration, epithelial injury, hyperplasia, goblet cell loss, and edema, providing a composite score of 0–4 (Supplementary Table 2) [17, 18]. For analysis of colon regions, colon length of fixed, Swiss-rolled colons was measured in NDP.view2 software from the end of the proximal colon transverse folds to the rectum, and bisected, such that the proximal half was defined as “mid colon” and the distal half as “distal colon.” For visualization of the mucosal bilayer, colons were harvested and fixed, unflushed, in methacarn (60% methanol, 30% acetic acid, and 10% chloroform), followed by periodic acid-Schiff (PAS) staining (Sigma, 395B-1KT). Flushed formalin-fixed colons were stained with PAS using the same protocol to identify goblet cells.

### Cytokine ELISAs

Serum levels of TNF-alpha and IL-17 were quantified by ELISA (Biotechne, MTA00B-1 and M1700-1). Each sample O.D. value was calculated using 450 nm minus 570 nm values, and concentration (pg/mL) determined using a standard curve.

### RNA Sequencing

Colon regions were defined as previously described [11], with distal defined as the 3 cm portion starting from the rectum and midproximal (MP) defined as the 2 cm portion distal to the transverse folds (Supplementary Figure 1). For RNA isolation from colon tissue, colons were flushed, sectioned into regions, and flash frozen. For RNA isolation from CD45 + enriched cells, colon regions were mechanically and enzymatically digested and separated into single cells using 0.1 mg/mL DNAse1 (Sigma-Aldrich, 10 104 159 001) and 400 U/mL Liberase (Sigma-Aldrich, 05 401 119 001). Leukocytes were isolated using a Percoll gradient (GE Healthcare Life Science, 17089102); CD45 + cells were enriched using the Miltenyi QuadroMACS Separator (130-090-976), LS columns (130-042-401), and CD45 microbeads (130-052-301). RNA was isolated with the Invitrogen PureLink RNA Mini (12183020) or Micro kit (12183016) and treated with DNase (12185010).

Libraries were generated using NEB Next Ultra RNA Library Prep Kit for Illumina. Paired-end 150-bp sequencing was performed on an Illumina Novaseq 6000. Fastq files were submitted to Salmon (v1.9) [19] for gene level quantification using mouse transcriptome reference mm10 index (retrieved from refgenomes.databio.org). Raw counts per gene were aggregated across samples and submitted for immune cell profiling using CIBERSORT (v1.0) [20] against a mouse-specific reference matrix [21]. DESeq2 (v1.34) was performed for normalization of gene counts and differential expression at the gene level emphasizing FDR-adjusted *P* values. Normalized counts were analyzed using principal components analysis (*prcomp* in R) with *Z*-score centering and scaling of gene features per dataset. Utilizing mouse-associated hallmark gene sets from the R package msigdbr (v7.5.1), gene set enrichment analysis (GSEA) was performed using the fgsea (v1.20) R package.

### Leukocyte Isolation and Flow Cytometry

To isolate intra-epithelial leukocytes, colons flushed with cold calcium-magnesium-free solution and sectioned by region were mechanically and enzymatically digested (as above). Cells were stained with CD45, Ly6C, Ly6G, F4/80, and I-A/E antibodies, and myeloid populations characterized as previously described [11]. Cells were sorted using a BD FACSAria FUSION cell sorter: CD45 ^+^ CD11b^hi^Ly6C^hi^Ly6G^−^F4/80^−/lowI−^A/E^−/low^ (monocytic immature myeloid cells; MO-IMCs); CD45 ^+^ CD11b^hi^ Ly6C^int^Ly6G ^+^ F4/80^−^I-A/E^−^ (polymorphonuclear immature myeloid cells; PMN-IMC); CD45 ^+^ CD11b ^+^ Ly6G^−^Ly6C^−^F4/80^high^I-A/E^high^ (F4/80^high^I-E/A^high^); and CD45 ^+^ CD11b ^+^ Ly6G^−^Ly6C^−^F4/80^low^I-A/E^low^ (F4/80^low^I-A/E^low^).

### Statistical Analysis

For between group comparisons of continuous or categorical data, groups were analyzed using a 2-tailed non-parametric Mann–Whitney test. For comparisons of inflammation scores between colon regions of each mouse, analysis used a 2-tailed, paired, non-parametric Wilcoxon matched-pairs signed rank test. For between group comparisons of histopathologic inflammation subcategories, a Fischer's exact test was performed. Differences with a *P* value of ≤.05 were considered significant.

## Supplementary Material

jiag247_Supplementary_Data

## Figures and Tables

**Figure 1.**
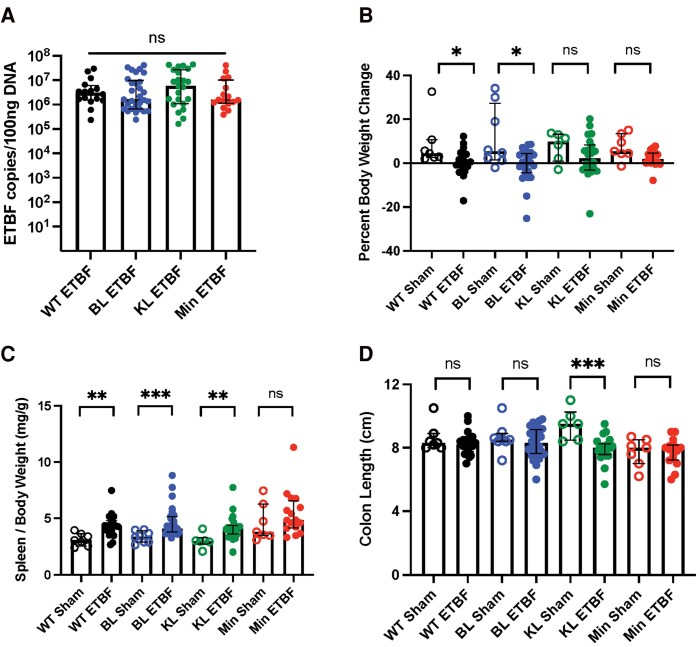
Colonization and clinical outcomes after 7 d of colonization with ETBF. *A*, Fecal colonization plotted as ETBF genome copies/100 ng of stool DNA. *B*, Body weights were measured at days 0 and 7. Data are depicted as percent change in body weight. *C*, Weights of harvested spleens (mg) respective to final body weights (g), depicted as a ratio. *D*, Length of unflushed colon (cm). In all panels, open circles represent mice gavaged with PBS (sham), while filled circles are indicative of the ETBF inoculated group in WT (black), BL (blue), KL (green), and Min (red) mice. Columns and error bars indicate median and interquartile range, respectively. Statistical significance was calculated by Mann–Whitney test, with *P* values presented as follows: * ≤.05, ** ≤.01, *** ≤.001, ns = not significant. N = 7–9 sham mice and 16–29 ETBF-treated mice per genotype from an aggregate of 8 independent experiments. See Fig S2 for confirmation of ETBF colonization by CFUs/g stool and serum cytokine ELISAs.

**Figure 2.**
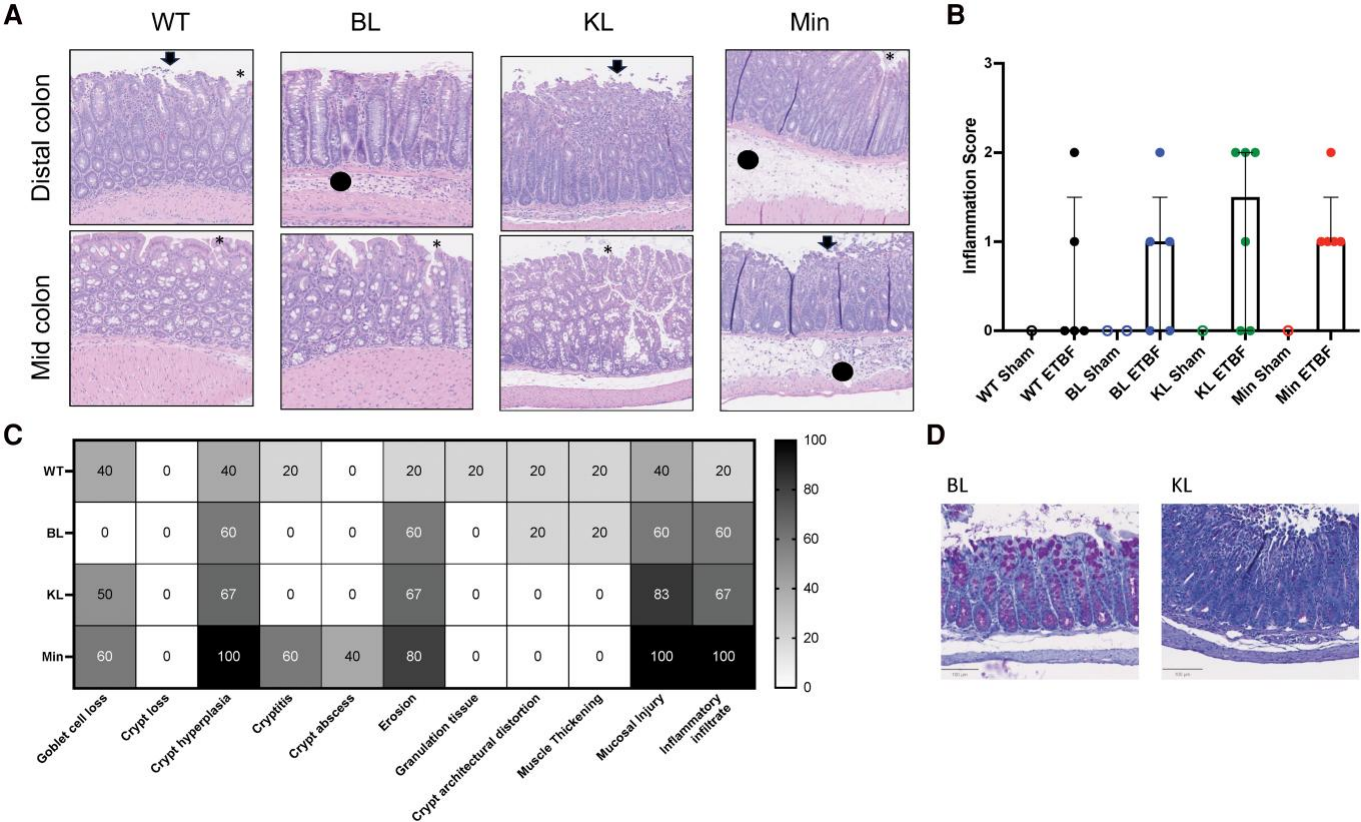
Host gene specific patterns of epithelial damage. *A*, Representative H&E-stained slides of distal and mid colon regions of formalin-fixed colons (see Methods for description of regions). Asterisks indicate crypt hyperplasia. Arrows indicate erosion. Black circles indicate submucosal expansion. *B*, Slides were scored by a blinded board-certified pathologist and assigned an inflammation score ranging from 0 to 4. Open circles represent mice gavaged with PBS (sham), while filled circles are indicative of the ETBF inoculated group in WT (black), BL (blue), KL (green), and Min (red) mice. Columns and error bars indicate median and interquartile range, respectively. N = 1–2 sham mice and 5–6 ETBF-treated mice per genotype from an aggregate of 2 independent experiments. See Fig S2D for regional inflammation scores by genotype. *C*, Categories of epithelial injury in ETBF-treated mice that contributed to the composite score in B, displayed as a heatmap depicting the percent of mice in each group who displayed the indicated pathologic findings. As indicated by the scale bar, the percent of affected mice per group ranges from 0% (white) to 100% (black). *D*, Representative PAS stained distal colons of ETBF-treated BL and KL mice to demonstrate differences in the degree of goblet cell loss. Scale bar = 100 μM.

**Figure 3.**
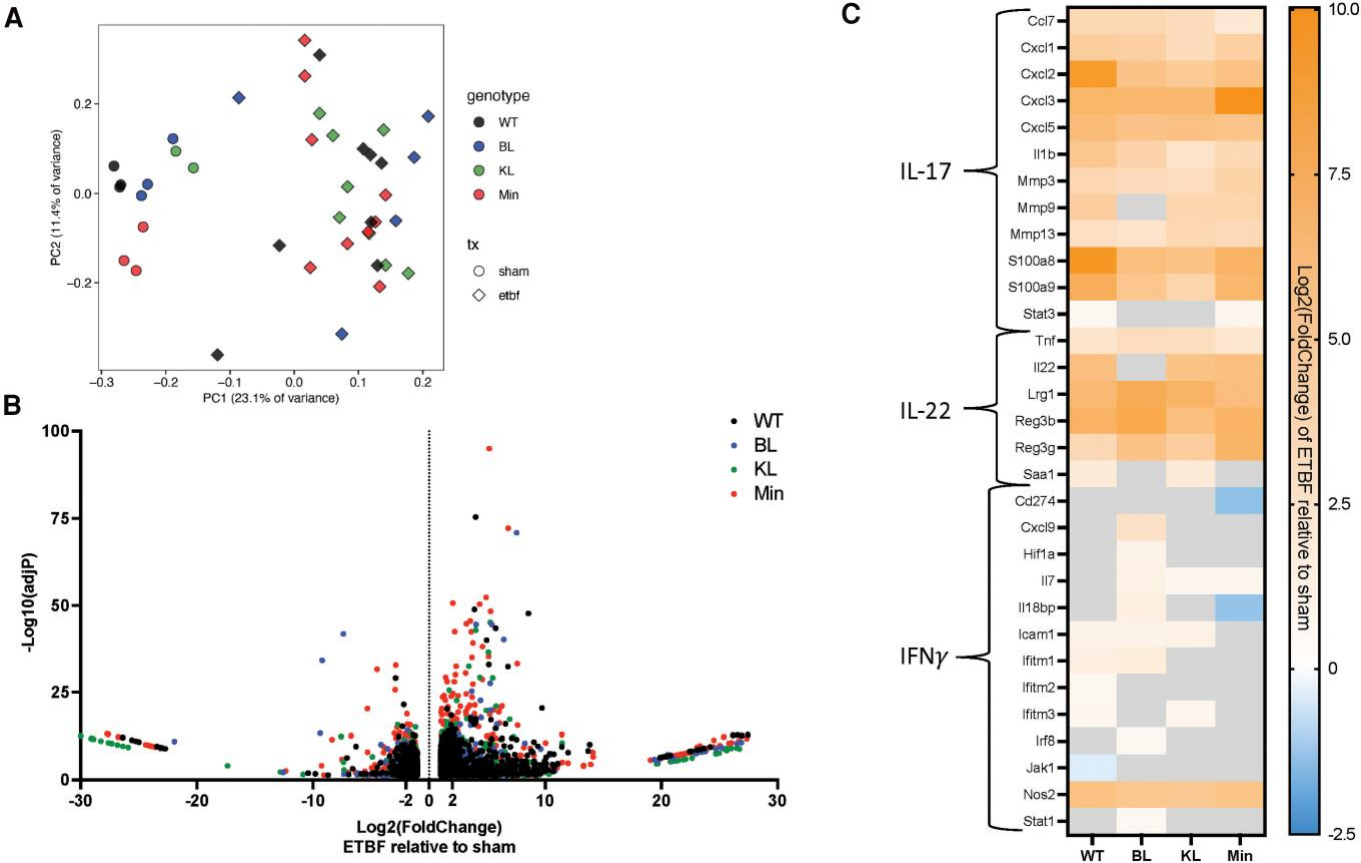
Host genotype-dependent changes in gene expression. RNA sequencing was performed on colon tissue from ETBF or sham-treated mice. *A*, Principal coordinates analysis (PCA) of distal colon RNA isolated from sham and ETBF-colonized WT (black), BL (blue), KL (green) and Min (red) mice. *B*, Volcano plot of differentially expressed genes by DESeq2 in WT (black), BL (blue), KL (green), and Min (red) ETBF-treated mice relative to their respective sham controls. All genes depicted are significantly upregulated (*P*Adj≤.05) in the ETBF-treated relative to sham (values >0) or downregulated in ETBF relative to sham (values <0). *C*, Relative expression of select genes associated with each indicated immune signaling pathway. For each gene, the Log_2_ (Fold Change) is depicted for ETBF-treated mice relative to sham for each genotype: significantly upregulated (orange) or downregulated (blue). Comparisons not reaching statistical significance (defined as *P*Adj≤.05) are shaded in gray. N = 2–3 sham mice per group and 5–9 ETBF-treated mice per group from an aggregate of 4 independent experiments.

**Figure 4.**
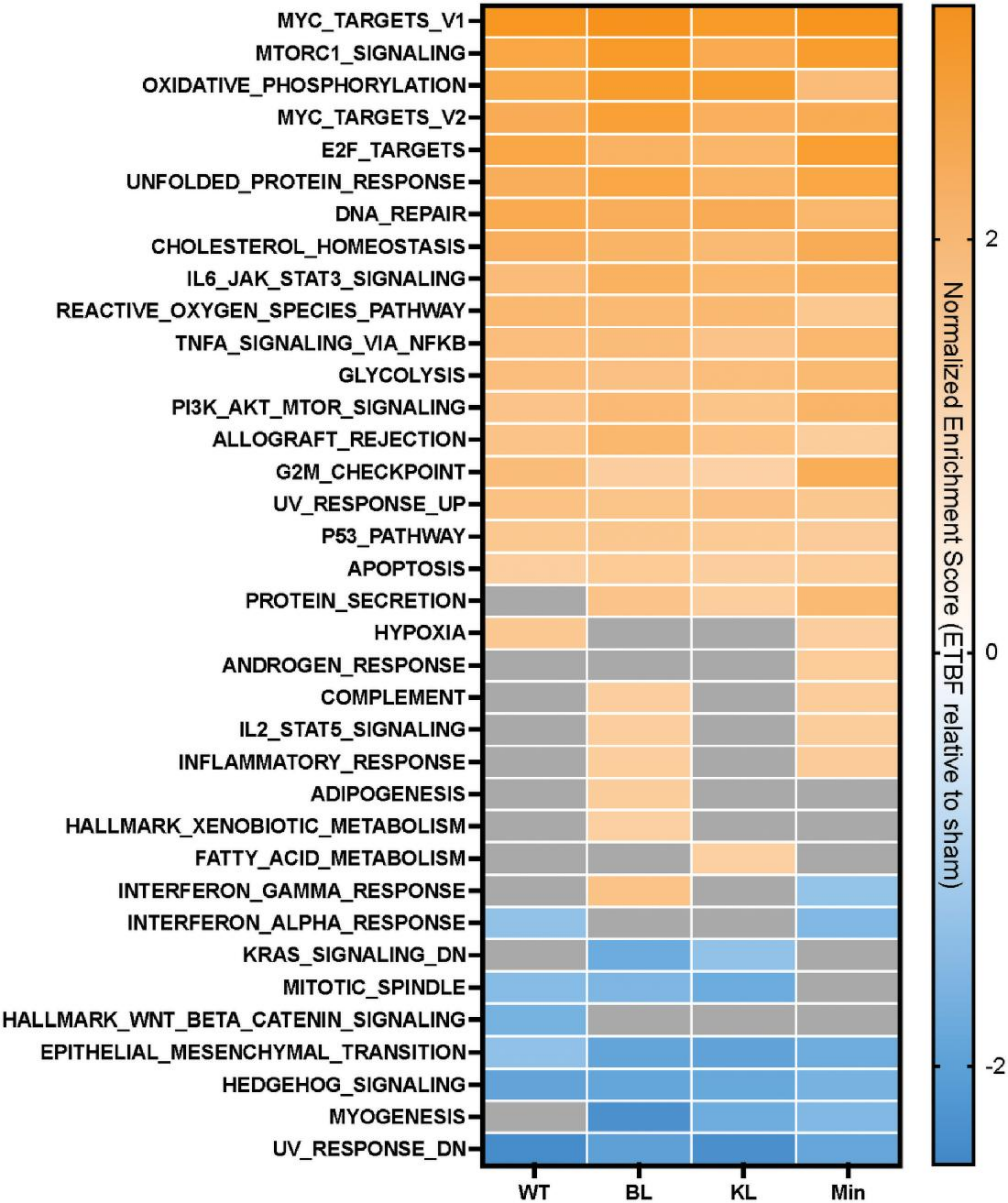
Host genotype-dependent changes in gene expression. Gene set enrichment analysis (GSEA) of distal colons of ETBF-treated mice relative to sham controls. For each labeled hallmark gene set, normalized enrichment scores (NES) are depicted significantly upregulated (orange) or downregulated (blue) in ETBF-treated mice relative to sham for each genotype. Comparisons not reaching statistical significance (*P*Adj≤.05) are shaded in gray. N = 2–3 sham mice per group and 5–9 ETBF-treated mice per group from an aggregate of 4 independent experiments. See Supplementary Figure 4 for individual gene set enrichment results for each genotype.

**Figure 5.**
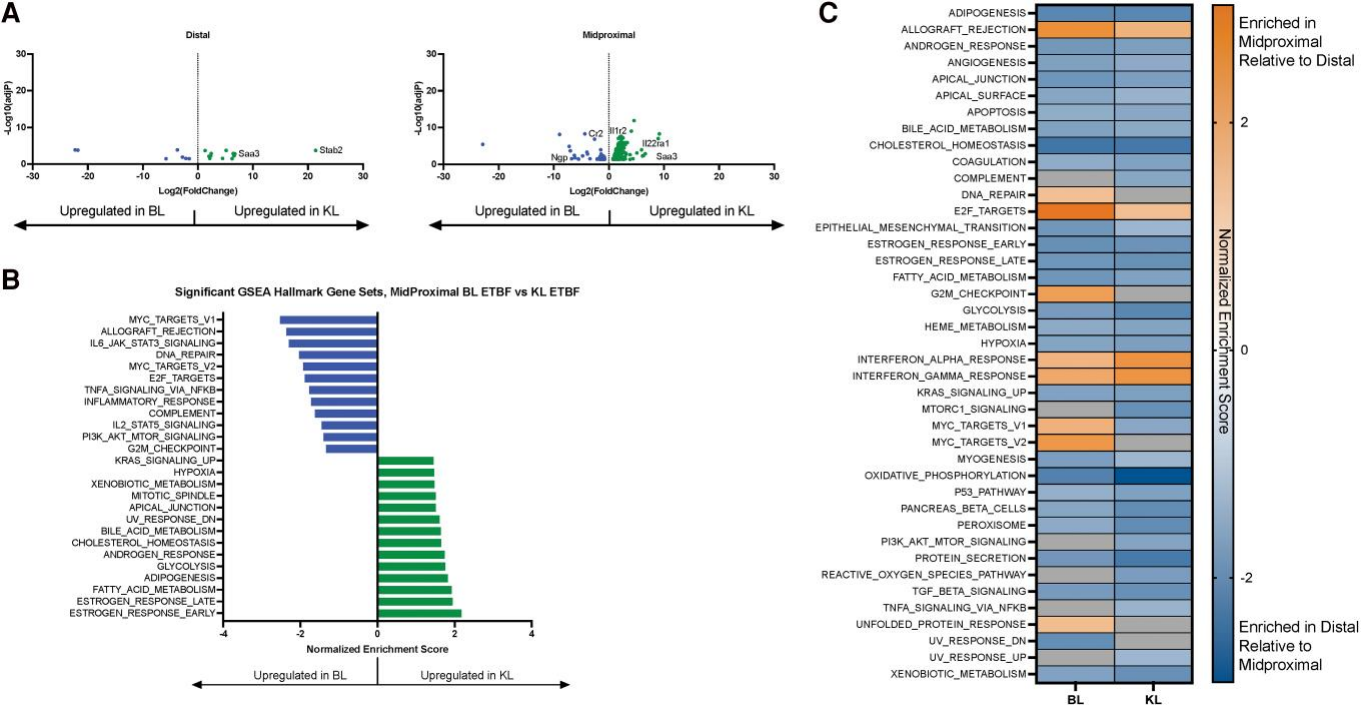
Colon region-specific changes in gene expression. RNA sequencing was performed on leukocyte (CD45+) enriched cell preparations from the distal or midproximal colons of ETBF-treated BL and KL mice. *A*, Volcano plots of differentially expressed genes by DESeq2 in the distal or midproximal colon cell preparations of ETBF-treated BL (blue) and KL (green) mice. All genes depicted are significantly upregulated (pAdj ≤0.05) in the KL groups relative to BL (values greater than 0) or upregulated in BL groups relative to KL (values less than 0). *B*, Hallmark gene sets significantly different between midproximal colons of BL and KL (*P*Adj≤0.05). *C*, Heat map of gene set enrichment analysis (GSEA) of ETBF-treated BL and KL mice with expression in distal colon relative to midproximal colon samples. For each labeled hallmark gene set, normalized enrichment scores (NES) are depicted significantly upregulated in midproximal (orange) or upregulated in distal (blue), with those not reaching a significance value of *P*Adj≤.05 depicted in gray. N = 3 mice per group.

**Figure 6.**
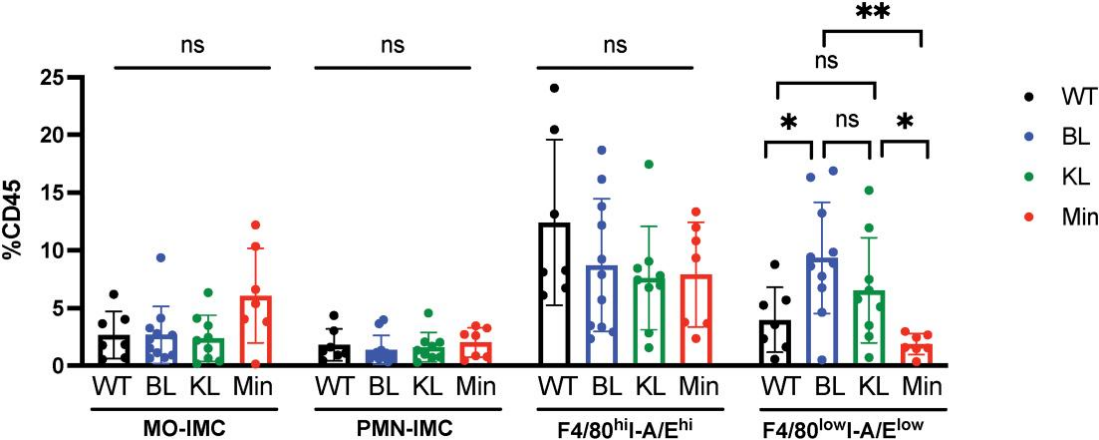
Myeloid Recruitment into the midproximal colon. Myeloid populations in the midproximal colon of ETBF-treated mice sorted by flow cytometry. Cells were stained and gated as follows: Immature monocytic cells (MO-IMC): CD45 ^+^ CD3^−^CD11b ^+^ Ly6C^hi^Ly6G^−^F4/80^−/low^/I−A/E^−/low^. Immature polymorphonuclear cells (PMN- IMC): CD45 ^+^ CD3^−^CD11b ^+^ Ly6C^int^Ly6G ^+^ F4/80^−^I-A/E^−^. CD45 ^+^ CD11b ^+^ Ly6G^−^Ly6C^−^ cells were further subsetted into F4/80^high^I-A/E^high^ and F4/80^low^I-A/E^low^. Between group comparisons for all data were analyzed by Mann–Whitney U test, with *P* values presented as follows: * ≤.05, ** ≤.01, *** ≤.001, ns = not significant. N = 7–9 mice per group from an aggregate of 5 independent experiments.

## Data Availability

These data have been made publicly available in accordance with the NIH Data Management and Sharing Policy. The data underlying this article are available in the article and its online Supplementary material, with the exception of raw and processed sequencing data, which is available in the NCBI Geo database at https://www.ncbi.nlm.nih.gov/gds/and can be accessed with accession numbers GSE327695 and GSE327693.

## RESULTS

### Enterotoxigenic *Bacteroides fragilis* Promotes Systemic Illness

Following administration of broad-spectrum antibiotics, a single orogastric gavage of ETBF is known to yield uniform colonization and acute colitis predominantly in the distal colons of C57BL/6 (WT) and *Apc^Δ716/+^* (Min) mice [8, 22]. It was previously demonstrated that *BRAF^−V600E^Lgr5^Cre^* (BL) mice developed marked colitis in both the distal and midproximal colon following ETBF colonization [11]. We sought to compare WT, Min, and BL mice with *Kras^G12D^Lgr5^Cre^* (KL) mice 7 days after ETBF gavage. This time point is representative of early host responses to ETBF, after stable ETBF colonization is established and myeloid populations are recruited to the colon, but before the formation of microadenomas in Min mice [8, 11, 23].

ETBF colonization was assessed in fecal pellets by qPCR (Figure 1*A*) and confirmed by enumeration of CFUs in a subset of mice (Supplementary Figure 2A), demonstrating similar ETBF fecal colonization regardless of genotype. Seven days after gavage, WT and BL mice colonized with ETBF had slower growth in body weight from day 0 to 7 relative to PBS-gavaged sham animals(*P* = .02), with a trend toward lower percent body weight change in Min mice (*P* = .05) (Figure 1*B*). BL mice had higher serum concentrations of TNF-α compared with WT and KL (*P* = .05), with no differences in IL-17 (Supplementary Figure 2B–C). Enterotoxigenic *Bacteroides fragilis*-colonized WT, BL, and KL mice displayed splenomegaly relative to shams (*P* = .005, .0008, and .003, respectively), suggestive of a systemic immune response (Figure 1*C*). Min mice, meanwhile, had comparable spleen weights whether treated with ETBF or PBS. Enterotoxigenic *Bacteroides fragilis*-treated KL mice had significantly shorter colons than shams (*P* = .0004) (Figure 1*D*), a possible indicator of acute colitis [24]. These data suggest that host genotype may alter both local and systemic responses to ETBF colonization.

### Enterotoxigenic *Bacteroides fragilis* Induces Host Gene Specific Patterns of Epithelial Damage

To further characterize colonic inflammation, we assessed H&E-stained colon tissue for evidence of epithelial damage and acute inflammation (Figure 2*A*). Enterotoxigenic *Bacteroides fragilis*-colonized mice of all genotypes had similar inflammation scores in sum (Figure 2*B*). There were trends toward different patterns of epithelial changes, alterations in mucosal architecture, and distribution of inflammation (Figure 2*C*), although none reached statistical significance. Enterotoxigenic *Bacteroides fragilis*-colonized mice of all genotypes displayed crypt hyperplasia, mucosal injury, inflammatory infiltrate, and areas of focal erosion. Interestingly, cryptitis and crypt abscesses were common in Min mice, but not visualized in any BL or KL mice, and only rarely in WT mice. Several WT and BL mice displayed crypt architectural distortion and muscle thickening, whereas no KL or Min mice had these findings. Notably, BL mice were the only group not to display goblet cell loss in response to ETBF, a prominent finding in the other genotypes (Figure 2*C*–*D*). To investigate colon regional inflammation, we used the same rubric to assess inflammation scores in the mid and distal colon, and found no statistically significant differences in inflammation scores between colon regions for any genotype (Supplementary Figure 2D).

### Enterotoxigenic *Bacteroides fragilis* Induces Host Genotype-Dependent and Colon Region-Specific Changes in Gene Expression.

We next performed bulk RNA sequencing on distal colon tissue of ETBF- or sham-treated mice at day 7 (Figure 3). Samples primarily clustered by treatment type, with samples clustered by genotype within the sham group (Figure 3*A*). Differential expression analysis of sham colons revealed baseline patterns of gene expression that may reflect distinct colonic epithelial functions that are oncogene dependent (Supplementary Table 3). For example, BL and KL mice had higher baseline expression of *muc2* and *muc16* compared with WT and Min animals. *Muc4* expression followed similar patterns, although not significant for BL mice (Supplementary Figure 3A). It was previously demonstrated that mice expressing *BRAF^V600^*^E^ had increased expression of *muc2* and a disorganized mucus bilayer in the midproximal colon [11]. To assess whether altered expression of mucins corresponded with altered mucus bilayer structure, we performed PAS staining of colons from sham animals (Supplementary Figure 3B). Consistent with previous observations, BL mice had disruption of the normal mucus bilayer in the midproximal colon, not observed in the other genotypes.

When ETBF and sham groups were compared for each genotype, many of the same genes had increased or decreased expression in response to ETBF regardless of genotype (Figure 3*B*, Supplementary Table 3). All genotypes demonstrated significant upregulation of proinflammatory chemokine and cytokine genes involved in IL-17 signaling and IL-22 signaling (Figure 3*C*). Similarly, a high degree of consistency was observed in GSEA hallmark gene set enrichment scores among all genotypes of ETBF-colonized mice including pathways involved in inflammation, such as TNFα signaling via NFκB, IL6/JAK/STAT3 signaling, and reactive oxygen species pathways (Figure 4, Supplementary Figure 4, Supplementary Table 4). All genotypes had enrichment of DNA repair, MYC Targets V1 and V2, E2F Targets, P53 Pathway, and P13 K/AKT/MTOR pathways involved in initiation of pro-carcinogenic pathways. Hedgehog and Epithelial-Mesenchymal Transition pathways, meanwhile, were downregulated in response to ETBF for all genotypes.

However, there were many notable genes and pathways where altered expression in response to ETBF correlated with host genotype (Figure 3*B*–*C*, Figure 4). For instance, Complement, IL2/STAT5 Signaling, and Inflammatory Response pathways were upregulated in response to ETBF only in BL and Min mice. We were most interested in identifying gene expression differences between BL and KL mice, to begin to understand the phenotypic differences in colon tumors when *BRAF^V600E^* and *Kras^G12D^* are combined with a loss of function mutation in *Apc* [11]. Both BL and KL animals had significant upregulation of *Il24* in response to ETBF (Supplementary Table 3), although this was more profound in BL (21-fold change) than KL mice (6.6-fold change). IL-24 is implicated in induction of a Th1 immune response [25], promoting IFNγ secretion from T cells, consistent with previous observations of the TiME of ETBF-induced colon tumors in *BRAF^V600E^* mice [11]. Likewise, the interferon gamma response pathway and several IFNγ response genes (eg, *Cxcl9*, *Il18 bp*, *Irf8*, and *Stat1*) were uniquely upregulated in BL mice. The Fatty Acid Metabolism pathway was uniquely upregulated in KL mice colonized with ETBF, as was the *Il33* gene. This tissue injury-associated cytokine was found to be upregulated in mouse models of acute colitis, where it has a protective role, enhancing epithelial barrier function [26].

We also analyzed leukocyte-enriched (CD45+) cell preparations from distal and midproximal colons of BL and KL mice (Supplementary Table 5). The purpose was 2-fold: (1) to enrich immune cells from our samples for genotype-specific study, and (2) to determine whether region-specific responses in BL and KL mice occur as has previously been defined in Min mice [22]. Overall, transcriptional profiles were remarkably similar between BL and KL distal mouse colons, whereas there were substantially more differentially expressed genes when midproximal colons were compared (Figure 5*A*; Supplementary Table 5). Analysis of hallmark gene sets (Figure 5*B*; Supplementary Table 6) from midproximal colon samples demonstrated enrichment of several pathways related to acute inflammation in ETBF-treated BL mice compared with KL mice (eg, IL6/Jak/STAT3 Signaling, TNFα Signaling via NFκB, Inflammatory Response, and IL2/STAT5 Signaling), as well as enrichment of several metabolic pathway gene sets in KL mice relative to BL (eg, Fatty Acid Metabolism, Bile Acid Metabolism, and Cholesterol Homeostasis). Analysis of hallmark gene sets further demonstrated regional differences in gene expression unique to each genotype (Figure 5*C*). BL colons had significantly higher enrichment scores in the midproximal colon for several gene sets related to tumorigenesis, including DNA Repair, E2F Targets, G2 M Checkpoint, Unfolded Protein Response, and Myc Targets 1 and 2. Both BL and KL mice had enrichment of Interferon Alpha and interferon gamma response pathways in the midproximal colon relative to distal. Meanwhile, Cibersort analysis failed to detect any statistically significant differences in immune cell signatures by genotype in either colon region (Supplementary Figure 5).

### Enterotoxigenic *Bacteroides fragilis* Promotes Recruitment of Distinct Myeloid Populations Dependent on Genotype

To further examine the immune response to ETBF, we characterized myeloid populations in the epithelium by flow cytometry. Min mice were previously found to accumulate monocytic MDSCs in response to ETBF, and this population was activated by IL-17 to promote tumorigenesis [23]. BL mice, in contrast, accumulate granulocytic MDSCs (PMN-MDSCs) in the midproximal, but not distal, colon after ETBF colonization [11]. This population, although expressing macrophage markers, has enrichment of genes associated with neutrophils, antibacterial enzymes, immunosuppressive molecules, and enzymes associated with T cell suppression [11, 27]. We sought to identify genotype-specific differences in the myeloid compartment of the midproximal colon after ETBF treatment. Consistent with previously published data [11], the midproximal colon of BL mice was enriched for a CD45 ^+^ CD11b ^+^ Ly6G^−^Ly6C^−^F4/80^low^I-A/E^low^ population compared with WT and Min mice (Figure 6). Although KL mice had significantly more F4/80^low^I-A/E^low^ cells than Min mice, there were no significant differences between KL and either WT or BL mice, suggesting an intermediate phenotype. Other myeloid populations (monocytic or neutrophilic immature myeloid cells and F4/80^hi^I-A/E^hi^ macrophages) did not differ between genotypes (Figure 6).

## DISCUSSION

Members of the colon microbiota such as enterotoxigenic *Bacteroides fragilis* (ETBF) have been implicated in colon tumorigenesis [6, 8]. However, little is known about whether oncogenes or tumor suppressor genes trigger distinct pathways of epithelial damage, inflammation, proliferation, and/or carcinogenesis dependent on specific microbiota members or communities. This is a critical gap in knowledge, as CRC has a diverse range of molecular and immune phenotypes with select driver mutations [28, 29]. While data suggest distinct microbiome composition in CRC biopsies of different molecular subtypes [30–33] and a linkage between host gene mutations and downstream microbiota changes [34], there are limited studies investigating functional impacts of the microbiota in tumors expressing distinct somatic mutations. Microbiota composition, structure, and function also appear to differ based on colon region [35–37]. Likewise, oncogenic mutations are associated with tumorigenesis in distinct colon regions [38, 39]. We propose that the microbiome and the host interact in distinctive ways along the colon axis, contributing to the development of colon tumors that are molecularly and immunologically diverse. As both the microbiome and oncogenic mutations are thought to drive tumorigenesis, understanding this interplay is of critical importance.

Previous investigations in mice expressing *Apc* mutations established that colonization with ETBF stimulates chronic colitis that promotes distal tumor formation through activation of IL-17, myeloid infiltration, and NFkB [8, 9]. Expression of *BRAF^V600E^* in this model alters tumor phenotype and immune responses [11]. These models underscore the impact of host genes on the proinflammatory and pro-carcinogenic signals triggered by oncomicrobes like ETBF, known to occur quickly in mice and largely within the first 2 weeks of colonization [40]. What remains unknown are the critical cells and signaling pathways that lead to these divergent phenotypes. We therefore chose to investigate ETBF-induced inflammation and cell signaling at 7 days postcolonization to examine the impact of clinically relevant oncogenic mutations.

Previous data demonstrate that regional differences in ETBF colonization alone do not account for genotype-specific differences in epithelial and immune responses [9, 11]. Our data suggest that CRC mutations alter host-bacteria interactions in ETBF colitis early in the disease process, perhaps owing in part to differences in the mucosal barrier. Mice expressing *BRAF^V600E^* lose the normal mucus bilayer architecture in the midproximal colon, but it is not yet understood whether and how this leads to altered barrier function. Does overproduction and disorganization of mucins change the composition of the mucus-adherent microbiota or its access to epithelial cells? These mice are robustly colonized with ETBF similar to other genotypes. However, while ETBF universally causes epithelial damage and inflammation, the nature of this damage differs in the presence of oncogenic mutations. *BRAF^V600E^* mice display a muc2- and goblet cell-rich phenotype, similar to certain human colon *BRAF^V600E^* tumors [41], whereas all other genotypes display goblet cell loss in response to ETBF colonization. In addition, ETBF promotes enhanced carcinogenic gene expression profiles in the midproximal colon of *BRAF^V600E^* mice, a unique locus of tumorigenesis in *BRAF^V600E^/Apc^Δ716^* mice [11].

ETBF pathogenicity results from the activity of the *Bacteroides fragilis* toxin (BFT), which acts exclusively on epithelial cells [8, 9, 22]. It is striking that a single bacterial toxin can exert such distinct effects depending on the host oncogenic landscape. We hypothesize that BFT exerts differential effects on the epithelium that subsequently trigger diverging immune responses. Enterotoxigenic *Bacteroides fragilis* pathogenicity in Min mice has been attributed to a pro-carcinogenic inflammatory cascade within the distal colon epithelium involving Th17, myeloid cells, and NFkB signaling [9]. While ETBF promoted IL-17 inflammatory signaling in all genotypes we tested, we identified divergent immune responses to colonization across groups. We confirmed the previous finding that ETBF promoted IFNγ signaling and infiltration of PMN-MDSCs in *BRAF^V600E^* mice [11], signatures reduced in *Kras^G12D^* mice and absent in *Apc^Δ716/+^* mice. This *BRAF^V600E^*-associated interferon response may be a key mediator of the host response to checkpoint inhibitor therapy, as an interferon-high immunophenotype was recently shown to be critical in predicting immunotherapy responsiveness in individuals with CRC [42]. Indeed, ETBF-colonized mice expressing *BRAF^V600E^* in combination with *Apc^Δ716^*, but not *Apc^Δ716^* alone, demonstrate therapeutic response to PD-L1 blockade [11]. We hypothesize that the influx of PMN-MDSCs and cytotoxic T cells into tumors in these mice reflects a TiME amenable to the therapeutic impacts of PD-L1 blockade. Future studies testing this hypothesis will require transcriptional and functional analysis of the tumor-infiltrating immune cells in mice co-expressing MAPK and *Apc* mutations, as the former is insufficient to facilitate microbially induced tumor formation in murine models to date [11, 43, 44].

These data highlight the importance of host cell gene context for understanding the contributions of the microbiota to disease pathogenesis. More investigations are needed to better elucidate the interplay between oncogenes, microbiota members, and the host immune response to lay the groundwork for a personalized medicine approach to cancer microbiome therapeutics.

## Supplementary Data

Supplementary materials are available at *The Journal of Infectious Diseases* online (http://jid.oxfordjournals.org/). Supplementary materials consist of data provided by the author that are published to benefit the reader. The posted materials are not copyedited. The contents of all supplementary data are the sole responsibility of the authors. Questions or messages regarding errors should be addressed to the author.
