# Supplementary material for: Enterotoxigenic Bacteroides fragilis Induces Host Genotype-Specific Colonic Epithelial and Immune Responses in Mice
Source: J Infect Dis. 2026 May 6;234(1):e110–20. doi: 10.1093/infdis/jiag247 (PMC13431769; doi:10.1093/infdis/jiag247)
Supplement: jiag247_Supplementary_Data [file jiag247_supplementary_data.zip › SouthwardZhuang_Supplemental Figure Legends.docx]

**Supplemental Figure Legends.
Figure S1. Diagram delineating regions of the colon.** Colons were bisected to visualize the gross anatomic architecture from the proximal colon to the rectum. Midproximal colon was defined as the 2cm portion distal to the transverse folds. Distal was defined as the 3cm portion starting from the rectum.

**Figure S2. ETBF colonization and systemic response.** (A) Colonization of ETBF in WT, BL, KL and Min mice as determined by colony forming units (CFU) per gram of stool 7 days after oral gavage of ETBF. Serum levels of IL-17 (B) and TNF-𝜶 (C) in ETBF treated mice, as measured via ELISA. (D) Mid (triangle) and distal (square) colon regions from ETBF-treated mice were scored as in Fig 2 by a blinded board-certified pathologist and assigned an inflammation score ranging from 0-4. WT (black), BL (blue), KL (green), and Min (red) mice. Columns and error bars indicated median and interquartile range, respectively.

**Figure S3. Mucin expression and mucus layer architecture.** (A) Volcano plot of differentially expressed genes by DESeq2 in the distal colons of BL (blue), KL (green), and Min (red) sham mice relative to WT sham mice. Gene expression not reaching significance (defined as pAdj≤0.05) are depicted in gray. (B) Representative PAS staining of Methacarn-fixed, unflushed colons of sham-treated animals, with mouse genotype and colon region as denoted. Scale bar = 100 μM.

**Figure S4. Host genotype -dependent changes in gene expression.** Gene set enrichment analysis (GSEA) of distal colons of ETBF-treated mice relative to sham controls. For each labeled hallmark gene set, normalized enrichment scores (NES) >0 are significantly upregulated in ETBF-treated mice relative to sham controls. All genes depicted are significantly up- or down-regulated (pAdj≤0.05). N = 2-3 sham mice per group and 5-9 ETBF-treated mice per group from an aggregate of 4 independent experiments. See Fig 4 for a composite heatmap of this data.

**Figure S5. Cibersort predictions of genotype and colon region-specific immune populations.** Relative abundance of immune cell populations predicted by Cibersort Analysis of RNA isolated from midproximal and distal colon tissue enriched for CD45+ cells.

**Alt Text**

**Figure S1. Diagram delineating regions of the colon.** A diagram depicting the definition of midproximal and distal colon regions.

**Figure S2. ETBF colonization and systemic response.** A series of bar graphs depicting (A) Fecal colonization of ETBF as colony forming units (CFU) per gram of stool, (B and C) cytokine concentrations in serum, and (D) inflammation scores for colon regions by genotype.

**Figure S3. Mucin expression and mucus layer architecture.** (A) Volcano plot of differentially expressed genes by DESeq2 in the distal colons of sham mice from each genotype. (B) Representative PAS stained slides to visualize the mucus bilayer.

**Figure S4. Host genotype -dependent changes in gene expression.** Bar graphs depicting gene set enrichment analysis (GSEA) of distal colons of ETBF-treated mice relative to sham controls, one for each genotype.

**Figure S5. Cibersort predictions of genotype and colon region-specific immune populations.** Bar graphs depicting the relative abundance of immune cell populations predicted by Cibersort Analysis of RNA isolated from midproximal and distal colon tissue enriched for CD45+ cells.
