# Supplementary figures and images for: Enterotoxigenic Bacteroides fragilis Induces Host Genotype-Specific Colonic Epithelial and Immune Responses in Mice
Source: J Infect Dis. 2026 May 6;234(1):e110–20. doi: 10.1093/infdis/jiag247 (PMC13431769; doi:10.1093/infdis/jiag247)

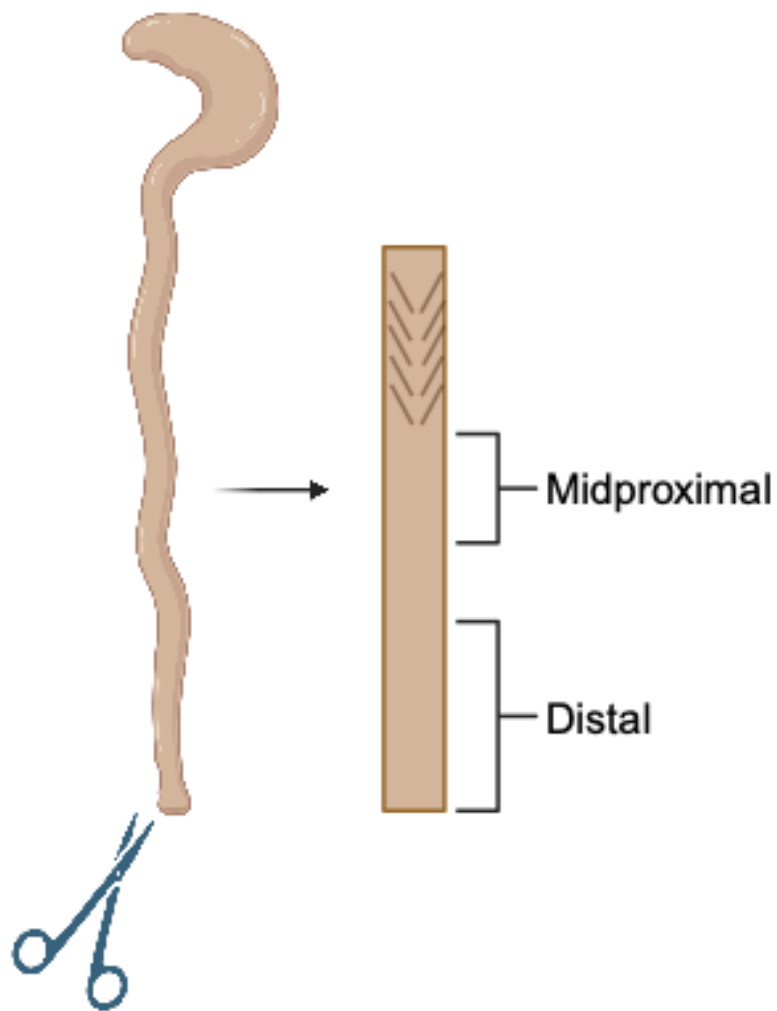

Supplement: jiag247_Supplementary_Data [file jiag247_supplementary_data.zip › Fig S1.pdf]

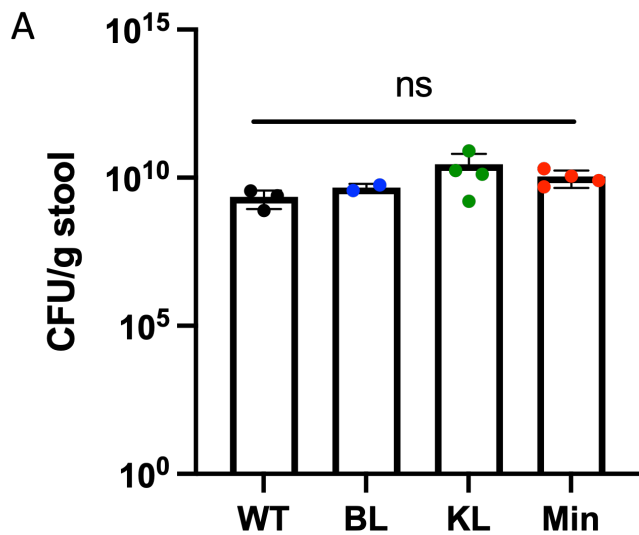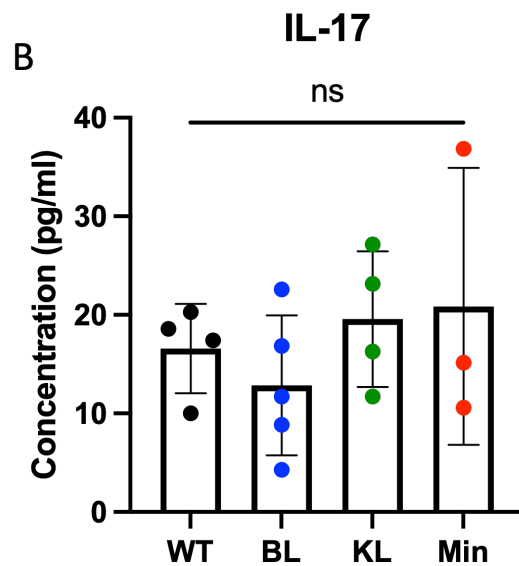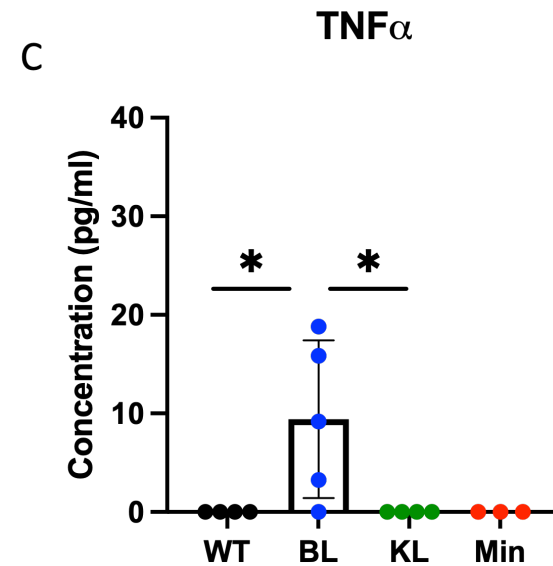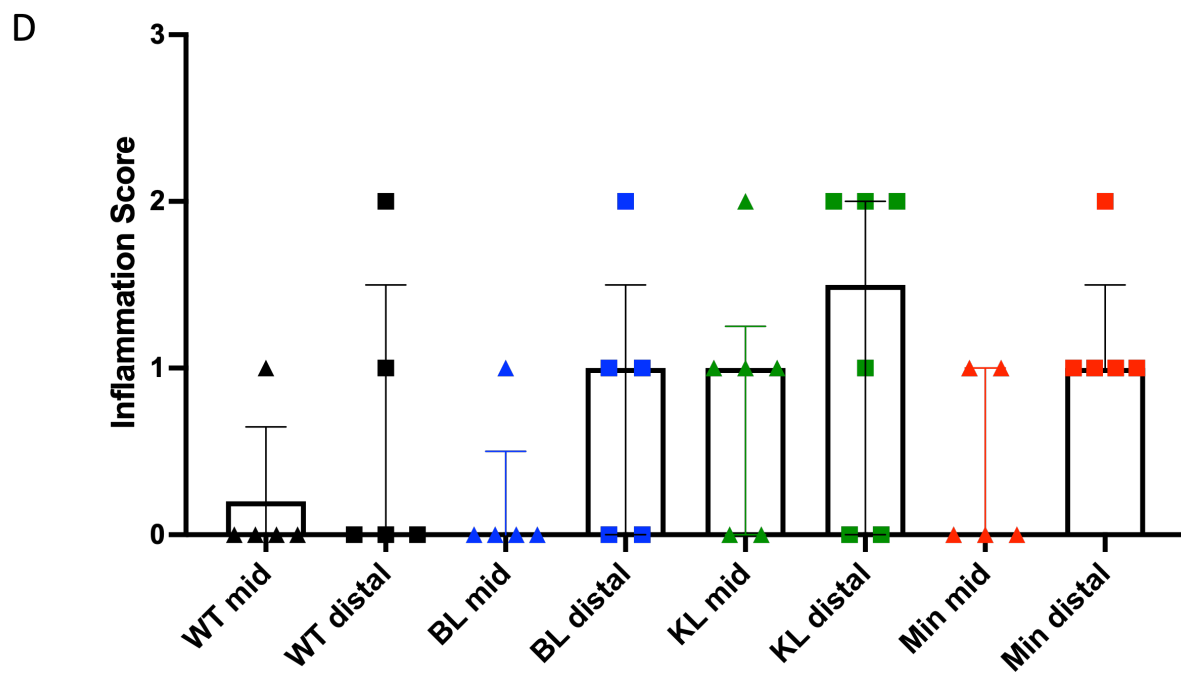

Supplement: jiag247_Supplementary_Data [file jiag247_supplementary_data.zip › Fig S2.pdf]

A

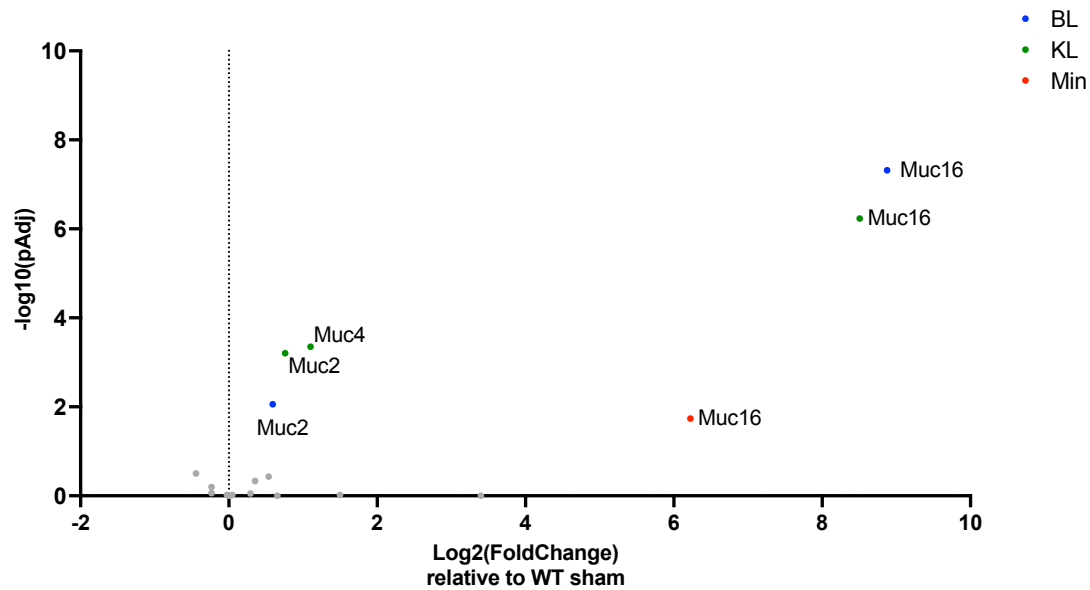

B

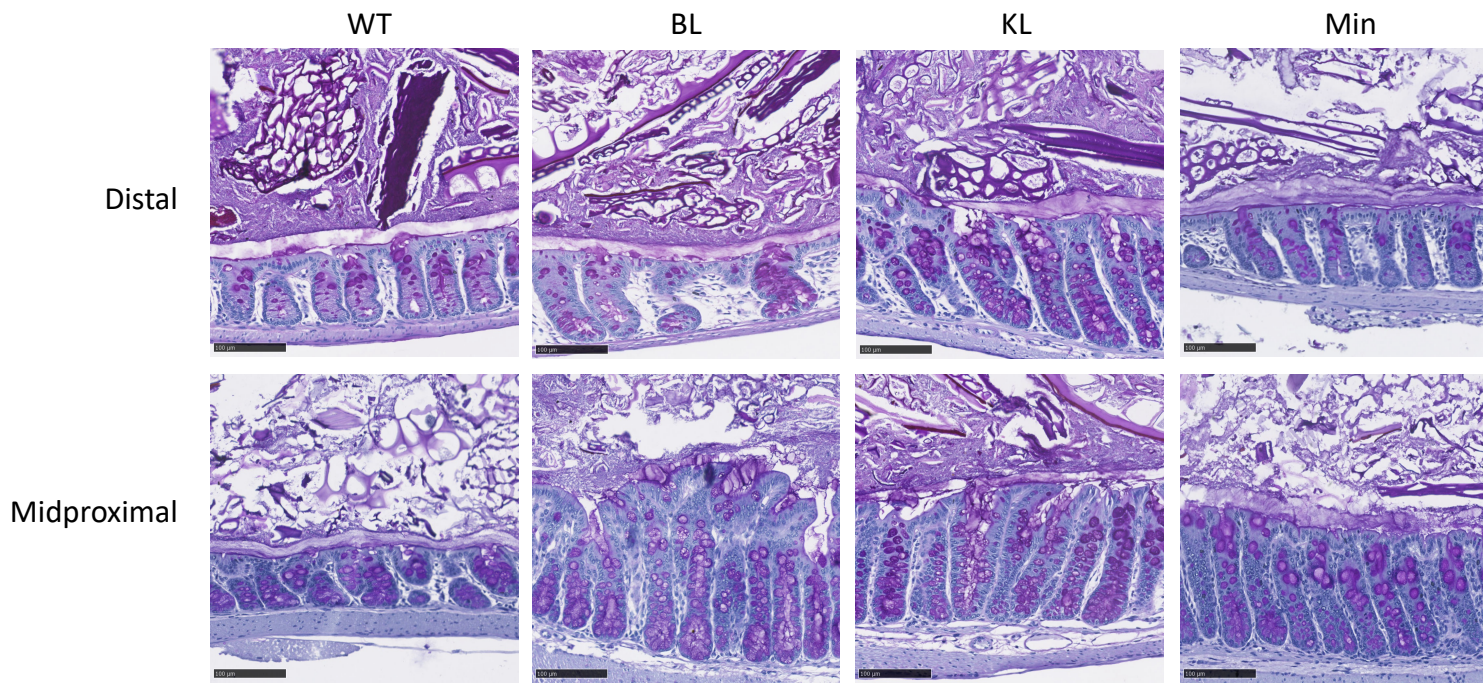

Supplement: jiag247_Supplementary_Data [file jiag247_supplementary_data.zip › Fig S3.pdf]

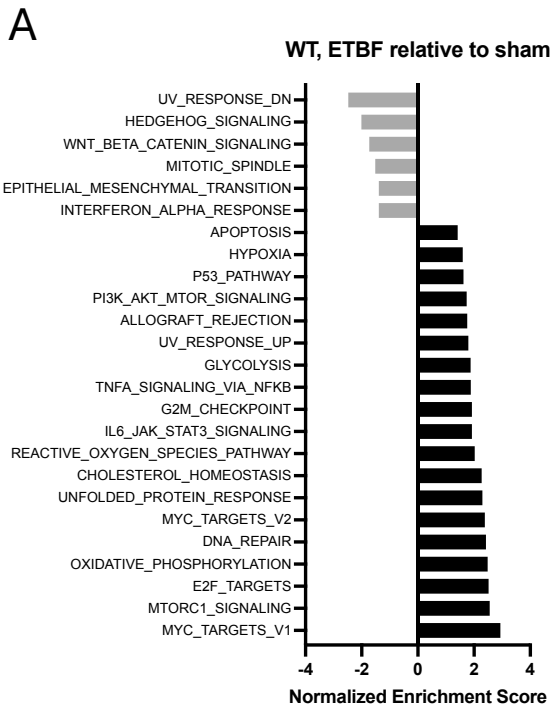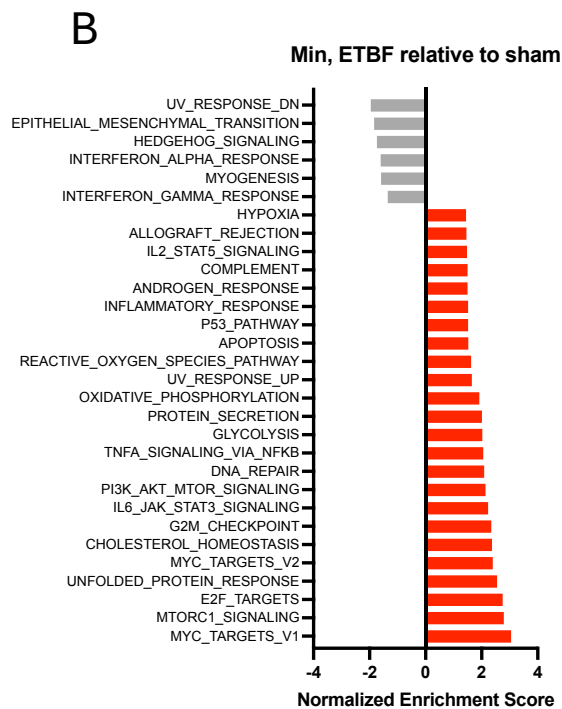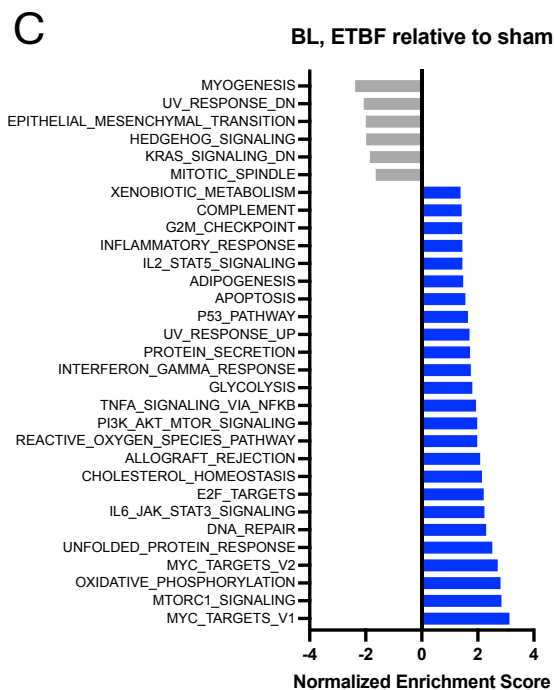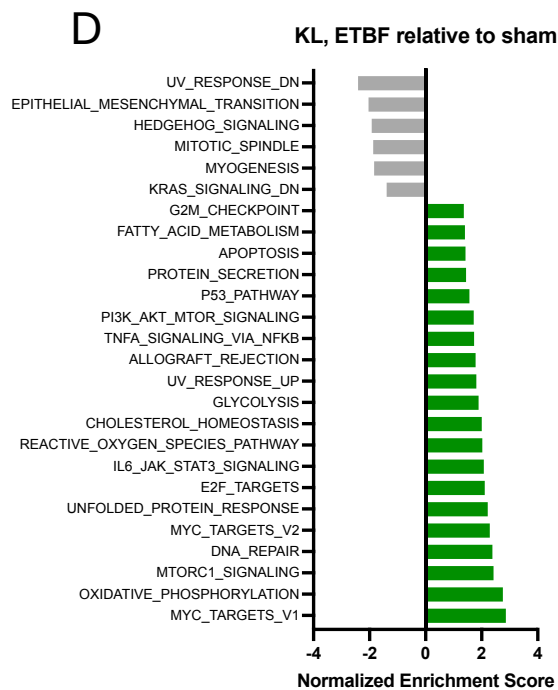

Supplement: jiag247_Supplementary_Data [file jiag247_supplementary_data.zip › Fig S4.pdf]

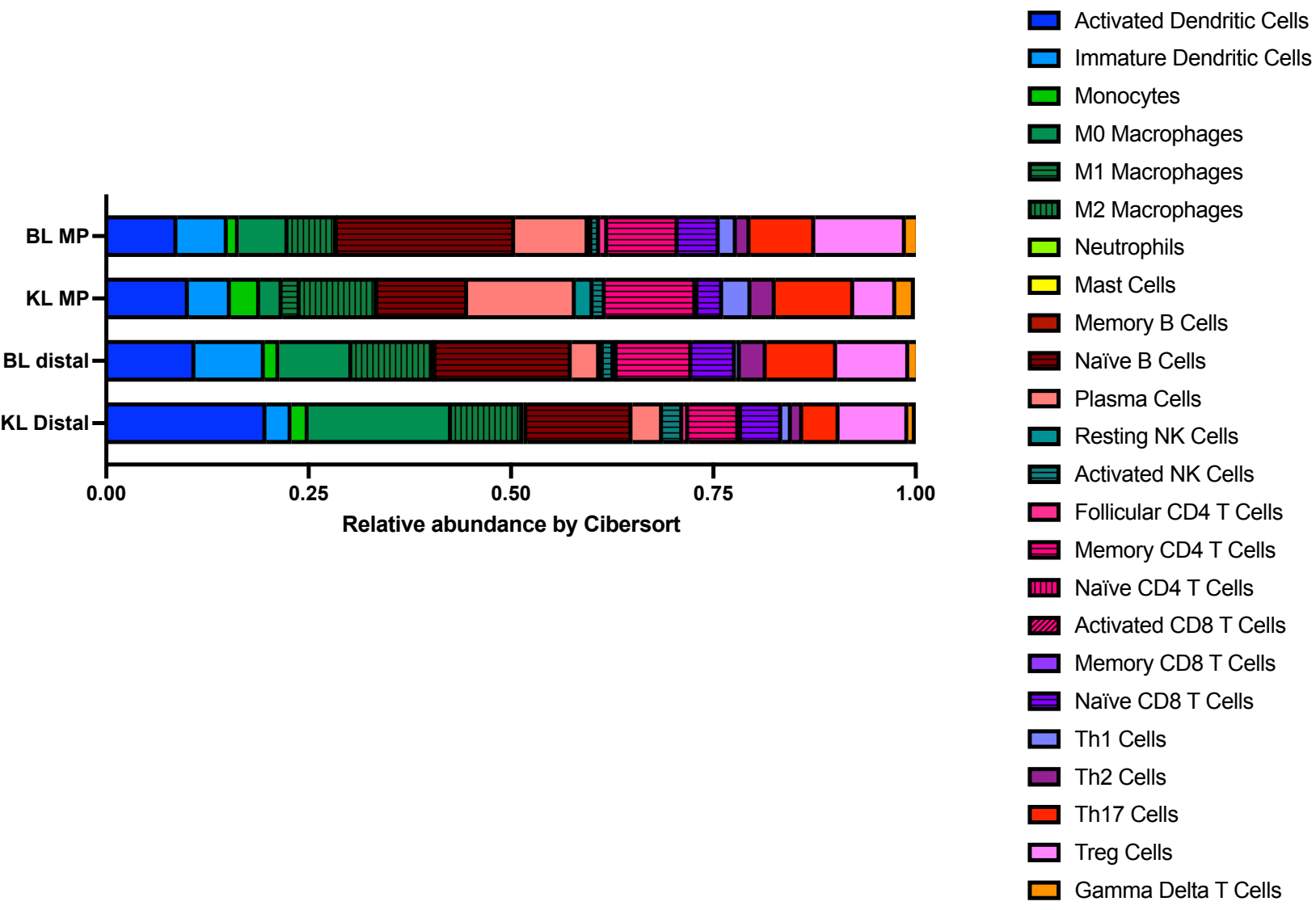

Supplement: jiag247_Supplementary_Data [file jiag247_supplementary_data.zip › Fig S5.pdf]
